# Diversified Carbohydrate-Binding Lectins from Marine Resources

**DOI:** 10.4061/2011/838914

**Published:** 2011-11-15

**Authors:** Tomohisa Ogawa, Mizuki Watanabe, Takako Naganuma, Koji Muramoto

**Affiliations:** Department of Biomolecular Sciences, Graduate School of Life Sciences, Tohoku University, Sendai 980-8577, Japan

## Abstract

Marine bioresources produce a great variety of specific and potent bioactive molecules including natural organic compounds such as fatty acids, polysaccharides, polyether, peptides, proteins, and enzymes. Lectins are also one of the promising candidates for useful therapeutic agents because they can recognize the specific carbohydrate structures such as proteoglycans, glycoproteins, and glycolipids, resulting in the regulation of various cells via glycoconjugates and their physiological and pathological phenomenon through the host-pathogen interactions and cell-cell communications. Here, we review the multiple lectins from marine resources including fishes and sea invertebrate in terms of their structure-activity relationships and molecular evolution. Especially, we focus on the unique structural properties and molecular evolution of C-type lectins, galectin, F-type lectin, and rhamnose-binding lectin families.

## 1. Introduction

Marine bioresources such as marine cyanobacteria, algae, invertebrate animals, and fishes produce a great variety of specific and potent bioactive molecules including natural organic compounds such as fatty acids, polysaccharides, polyether, peptides, proteins, and enzymes. To date, many researchers focused on the marine natural products and their various pharmacological functions to develop new potent drugs including antimicrobials, anti-human immunodeficiency virus (HIV), anticancer, and Alzheimer's therapeutics. There are several excellent reviews that described the potent therapeutic agents derived from marine resources and their medicinal applications [1–3]. In the drug discovery from natural resources, lectins are one of the promising candidates for useful therapeutic agents because carbohydrate structures such as proteoglycans, glycoproteins, and glycolipids have been implicated in certain cell types and their physiological and pathological functions including host-pathogen interactions and cell-cell communications. For example, griffithsin (GRFT), a lectin isolated from red algae *Griffithsia* sp., showed a strong anti-HIV activity with half maximal effective concentration (EC50) of 0.043–0.63 *μ*M via specific binding to gp120, which is envelop glycoprotein anchored to the HIV membrane and involved in viral entry into cells by recognition of CD4 [4]. Cyanovirin (CN-V) isolated from *Nostoc ellipsosporum* cyanobacteria has been reported as potent lectin with anti-HIV activity [5].

 Lectins are group of sugar-binding proteins except for antibodies and enzymes that recognize specific carbohydrate structures, resulting in the regulation of various cells via glycoconjugates. Thus, they can identify the cell types and cell development stages including embryonic stem (ES) and induced pluripotent stem (iPS) cells through histochemical applications, flow cytometry, and lectin microarrays [6] because cells often display altered surface glycoproteins and glycolipids depending on the physiological and pathological conditions. Lectins are widely distributed in all taxa from microbial organisms, plant, and animal and are involved in numerous cellular processes that depend on their specific recognition of complex carbohydrates. Based on the structural similarity of carbohydrate recognition domain (CRD) and their characteristics, animal lectins are classified into several categories: C-type lectins (CTLs), galectins, I-type lectins, pentraxins, P-type lectins, tachylectins, and so forth [7]. 

 Intensive investigations have been carried out to clarify the biochemical and physiological properties of humoral lectins of marine resources including marine cyanobacteria, algae, and invertebrates such as barnacles [8–10], sea urchins [11, 12], sea cucumbers [13–18], horseshoe crabs [19–30], tunicates [31–36], mollusks [37–39], and fishes [40–69] as shown in Table 1. For fish and sea invertebrate lectins, they could be mainly classified into CTLs, galectins, F-type lectins, and rhamnose binding lectin (RBL) families in addition to the Ricin-type, Lily-type, 6x  *β*-propeller/Tectonin-type lectins (Table 1). Here, we review these multiple lectins from marine resources including fishes and sea invertebrate in terms of their structure-activity relationships and molecular evolution. 

## 2. C-Type Lectin Family

 C-type lectins (CTLs) are one of major animal lectin family, of which members bind in a Ca^2+^-dependent fashion to mono- and oligosaccharides. They adopt generally multi-domain structures and contain one or more highly conserved CRD consisting of 115–130 amino acid residues [72], which has a unique mixed *α*~*β* topology [73]. In the presence of Ca^2+^, CTLs initiate a broad range of biological processes such as adhesion, endocytosis, and pathogen neutralization [74]. C-type lectin domain (CTLD) superfamily is a large group of extracellular proteins with conserved CRD sequences but different function including more than a thousand identified members, most of which lacking lectin activity. Since CTLD superfamily has been first classified into 7 groups (I to VII) by Drickamer in 1993 [75]; the classification of CTLD was revised in 2002 with additional seven groups (VIII to XIV) by Drickamer and Fadden [76]; and in 2005 with three new groups (XV to XVII) by Zelensky and Gready [77]. Group I contains four members, versican, aggrecan, neurocan, and brevican, which contain a proteoglycan core peptide and a single CTLD in the vicinity of the C-terminus and for which the name “lectican” has been proposed [78]. Group II CTLs have a single transmembrane domain, an extracellular carboxyl terminus, and a cytoplasmic amino terminus; typical examples are the hepatocyte asialoglycoprotein receptor (ASGR) [79], dendritic cell-specific intercellular adhesion molecule-3-grabbing nonintegrin (DC-SIGN), and macrophage receptors. Group III CTLs are collectins, which consist of an amino terminal collagen domain and a carboxyl terminal CTLD. They participate in the host defense mechanism through complement activation and include serum mannose binding protein (MBP) and pulmonary surfactant proteins [80]. Group IV CTLs, or selectins, are involved in the adhesive interaction between leukocytes and vascular endothelial cells such as L-selectin (leucocytes), E-selectin (endothelial cells), and P-selectin (platelets) [81]. Group V CTLs are involved in signal transduction; typical examples are the natural killer cell receptors [82] and the low-affinity IgE receptor (CD23) [83]. Group VI CTLs are type I transmembrane proteins consisting of a cysteine-rich domain, a fibronectin type II domain, and tandem CRDs. This group includes the macrophage cell surface mannose receptor and a dendritic cell surface molecule DEC-205 [84]. Group VII includes soluble single-CRD proteins. This group includes the pancreatic stone protein (PSP) isolated from pancreatic stones, lithostathine, which considered an inhibitor of calcite crystal growth [85–87]. Interestingly, CTLs isolated from marine invertebrates such as acorn barnacle hemolymph [9, 88] and pearl shells [38] have also been reported to have unique multiple functions in their biomineralization, that is, the inhibitory activity toward the crystal growth of calcium carbonate based on their high association constants to calcium ions. Furthermore, fish-specific CTLD proteins have been identified as the type II antifreeze proteins (AFPs), which inhibit the freezing by binding to ice in plasma of cold-water-living fish species including herring (*Clupea harengus*), rainbow smelt (*Osmerus mordax*), Japanese smelt (*Hypomesus nipponensis*), sea raven (*Hemitripterus americanus*), and longsnout poacher (*Brachyopsis rostratus*) [89–91]. 

 Zelensky and Gready (2004) have reported the genome-level analysis on CTLD superfamily in *Fugu rubripes* genome that demonstrate the divergence of all CTLD superfamily proteins except for two groups V and VII present in mammals [92]. They also identified fish-specific CTLDs, AFPs, and dual-CTLD proteins, in Fugu genome. CTLs are diversified and widely distributed in animal kingdom including fishes as unique structural motifs and functional domains, regardless of whether they possess the sugar-binding properties or not. 

## 3. Galectin Family

**Figure 1:**
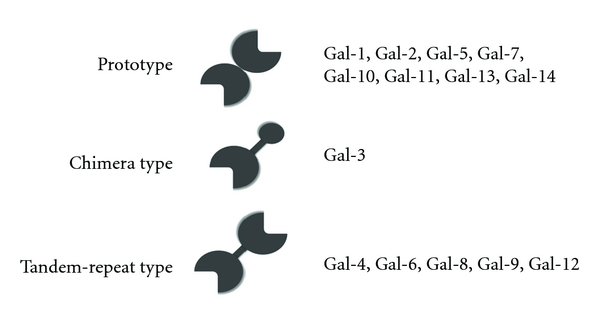
Classification of galectins.

Galectins are family of carbohydrate-binding proteins defined by their Ca^2+^-independent affinity for *β*-galactoside sugar, sharing a conserved sequence motif within their CRD of about 130 amino acid residues, the lack of a signal peptide and attached carbohydrate, and predominantly cytoplasmic location [99]. Based on the structural features, galectins can be classified into three types: prototype (monomer or homodimer of single carbohydrate-binding domain), tandem-repeat type (two carbohydrate-binding domains on a single chain), and chimera type (carbohydrate-binding domain and an extra N-terminal domain on a single chain) (Figure 1). Galectins have proposed to participate in diverse physiological phenomena such as development, differentiation, morphogenesis, immunity, apoptosis, metastasis of malignant cell, and so forth. To date, several galectins were identified from fishes including Japanese eel *Anguilla japonica* [40], electric eel *Electrophorus electricus* [44], channel catfish *Ictalurus punctuates* [53], windowpane flounder *Lophopsetta maculate* [49], zebrafish *Danio rerio* [50], and conger eel *Conger myriaster* [45–47]. 

**Figure 2:**
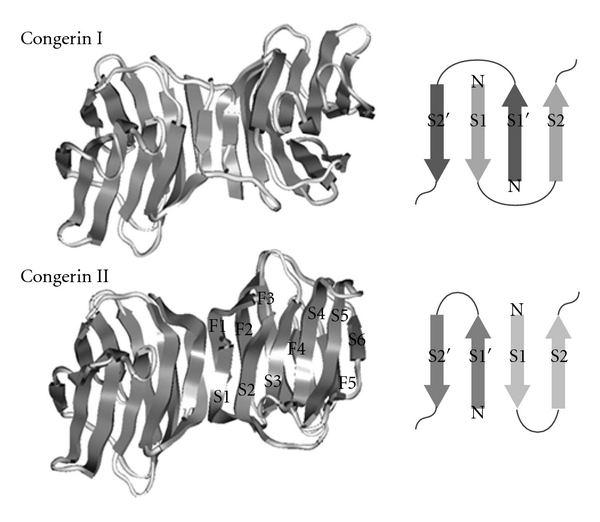
Schematic presentation of the wild-type congerin I (top: PDB code 1c1f) and congerin II (bottom: PDB code 1is5) and the topologies of **β**-sheet at subunit interface [70, 71].

Conger eel contains two prototype galectins, congerins I (Con I) and II (Con II). They are components of the biological defense system: these proteins mainly exist in the frontier organs and tissues that delineate the body from the outer environment, such as the epidermal club cells of the skin, wall of the oral cavity, pharynx, esophagus, and gills [46, 100], and agglutinate marine pathogen bacteria such as *Vibrio anguillarum* [101], and have opsonic and cytotoxic activities against cells [102–104]. It was reported that other fish galectin isolated from Japanese eel, AJL-1, also showed agglutinating activity against pathogenic Gram-positive bacteria, *S. difficile* [40]. Con I and Con II consist of 136 and 135 amino acid residues, respectively, and both contain several common structural characteristics: acetylated N-termini and no cysteine residue that is related to oxidizing inactivation found in mammalian galectins. Each subunit of Con I and Con II has one carbohydrate-binding site, and they form a homodimer to exhibit divalent crosslinking activity. Since a gene duplication event, these genes have been evolving at a high *dN*/*dS* (nonsynonymous/synonymous substitution) rate (2.6) under selection pressure, causing amino acid changes in Con I and Con II [46]. Usually, nonsynonymous substitutions in coding regions are restrained, by purifying selection, to maintain protein structure or function, whereas synonymous substitutions and nucleotide substitution for noncoding regions accumulate constantly by random genetic drift. Therefore, *dN*/*dS* ratios are normally smaller than 1.0 (*∼*0.2 in various genes). In other words, Con I and Con II have evolved via accelerated amino acid substitutions under positive selection. Similar evolutionary behavior has been observed only in several gene families including that of biological offense and defense systems such as snake venom isozymes and conus peptides and reproduction systems [105]. The structure of Con I demonstrated a protein-fold evolution by swapping *β*-strands between subunits, which altered the *β*-sheet topology at the dimer interface from entirely antiparallel to partially parallel to entangle two subunits, although Con I and Con II adopt the similar subunit structure with “jelly-roll” motif consisting of five-stranded and six-stranded *β*-sheets (Figure 2) [70, 71]. Domain swapping has been hypothesized as a mechanism in quaternary structure formation in protein evolution and reported several proteins [106, 107]. One of the important features of strand swapping seems to be increasing the stability of the quaternary structure, which is required for essential divalent cross-linking activity in agglutinating pathogenic bacteria (Figure 2). On the other hands, crystal structure analysis of Con II revealed that a MES (2-(*N*-morpholino) ethane sulfonic acid) molecule that corresponding to sulfonosugar bound to the cleft near the known CRD [71]. The similar extension of binding site toward the nonreducing end of lactose was observed in the fungal prototype galectins, ACG and CGL2 [108, 109]. Crystal structure of Con II complex with lacto-*N*-fucopentaose III at 2.2 Å resolution supported the extension of carbohydrate-binding site to the position where the MES was observed, indicating that the potent natural ligands of Con II include the additional moieties at the nonreducing end of lactose [110]. Actually, the differences in the thermostability and carbohydrate specificities between Con I and Con II were also observed [47]. Furthermore, to identify the determinants of selection pressures in the evolutionary process and the structural elements associated with the unique carbohydrate-binding activities of Con I and Con II, we recently reconstructed a probable ancestral form of congerin (Con-anc) that corresponded to the putative amino acid sequence at the divergence of Con I and Con II in the phylogenic tree [111, 112]. It was found that Con-anc showed the properties similar to those of Con II in terms of thermostability and carbohydrate-recognition specificity, although Con-anc shares the higher sequence similarity with Con I than Con II. From the differences between Con-anc and Con II on the sugar-binding specificities and mutation analysis of Con II, Con II has been considered to acquire the binding ability to the *α*2,3-sialyl galactose moieties such as GM3 and GD1a during the accelerated evolutionary event from Con-anc with the replacement of Arg3 and Tyr123 residues of Con II. The *α*2,3-sialyltransferases have been recently isolated and cloned from the pathogenic marine bacteria *Vibrio sp.* and *Photobacterium phosphoreum* [113, 114], suggesting that *α*2,3-sialyl galactose-containing sugars, which are presumed to be targets for Con II, may specifically be present in pathogenic marine bacteria. On the other hand, Con I has evolved from the ancestral congerin Con-anc to increase the binding activity against *α*1,4-fucosylated *N*-acetyl glucosamine [114]. These findings emphasize that the carbohydrate-binding ability and the specificities of congerins are diversified via accelerated evolution. 

 Cooper (2002) reviewed the genome-wide screening of galectin gene families (galectinomics) based on the genomic sequence database including *Arabidopsis*, *Drosophila*, *Caenorhabditis*, *Xenopus*, human, and zebrafish *Danio* [115]. In the zebrafish genome, homologues of mammalian galectins-1, 3, 4, 9, and HSPC159 were reported.  Table 2 summarized the results of searching for the homologues genes encoding galectins and related proteins in the eighth integrated assembly of the zebrafish genome, Zv8 (release 59, August 2010). In the zebrafish embryo, four proto-type galectin-1-like proteins, Drgal1-L1, Drgal1-L2, Drgal1-L3, and splice variant of Drgal1-L2, one chimera-type Drgal3, and two tandem-repeat galectins, Drgal9-L1 and Drgal9-L2, have been identified and characterized [50, 116]. They exhibited distinct phase-specific expression patterns during embryo development; for example, Drgal1-L1 is maternal; Drgal1-L2 is zygotic and expressed at postbud stage in notochord, while Drgal1-L3, Drgal3, Drgal9-L1, and Drgal9-L2 are both maternal and zygotic, and ubiquitously in adult tissues [50]. Furthermore, knock-down experiments in zebrafish embryo showed that Drgal1-L2 plays a key role in somatic cell differentiation through the skeletal muscle formation [117]. Zebrafish genes encoding proto-type galectins contain four exons with highly conserved exon-intron boundaries to that of mammalian galectin-1. Recently, the homologue of galectin-related interfiber protein (Grifin), which is a lens crystallin protein and one of galectin-related proteins (GRPs), has been also identified in zebrafish (DrGrifin) (Table 2), especially in the lens, particularly in the fiber cells of 2 days post fertilization embryos, and adult zebrafish tissues such as oocytes, brain, and intestine [118]. 

 Furthermore, novel galectin-related protein named CvGal, which contains four canonical galectin CRDs, has been discovered from the hemocytes of the eastern oyster, *Crassostrea virginica* [119]. CvGal can recognize both endogenous and exogenous ligands including bacteria, algae, and *Perkinsus* sp. as a soluble opsonin for pathogens or as a hemocyte surface receptor for both microbial pathogens and algae food ingested into digestive ducts and as a modulator for up-regulation of CvGal itself  [119]. Unique domain architecture for genes/proteins consist of galectin CRDs, nematogalectin, was also found in freshwater hydrozoan *Hydra* and marine hydrozoan *Clytia* [120]. Nematogalectin, a 28 kDa protein with an N-terminal GlyXY domain that can form a collagen triple helix followed by galectin CRD, is a major component of the nematocyst tubule and is transcribed by nematocyte-specific alternative splicing [120]. Thus, the galectin family proteins also diversified by unique evolutionary process including tandem duplication and accelerated evolution.

## 4. F-Type Lectin Family

F-type lectins (fucolectin), which bind fucose and share characteristic sequence motif, have been identified as immuno-recognition molecules in invertebrates and vertebrates such as horseshoe crab (*Tachypleus tridentatus*) [23] and Japanese eel (*Anguilla japonica*) [43]. The crystal structures of single CRD and tandem CRDs of F-type lectins with a jellyroll *β*-barrel topology have been reported for *Anguilla japonica* agglutinin (AAA) and MsaFBP32 from striped bass (*Morone saxatilis*), respectively, [121, 122]. Bianchet et al. described that the fold structure of AAA, F-type lectin motifs, is widely distributed in other proteins even with lower sequence similarities, for example, C1 and C2 repeats of blood coagulation factor V, C-terminal domain of sialidase, N-terminal domain of galactose oxidase, APC10/DOC1 ubiquitin ligase and XRCC1 [121]. Furthermore, it has been reported that the several proteins are homologous to or contained with F-type lectin CRDs, of which examples include *Streptococcus pneumoniae TIGR4*, furrowed receptor and CG9095 of *Drosophila melanogaster*, *Xenopus laevis* pentraxin 1 fusion protein, *Microbulbifer degradans *ZP_00065873.1, and yeast allantoises [121, 123] in addition to the tandem-repeated types of F-type lectins found in modern teleosts [64–66, 122], while F-type lectin CRD motifs are absent in genomes of higher vertebrates such as reptiles, birds, and mammals. 

## 5. Rhamnose-Binding Lectin Family

The rhamnose-binding lectins (RBLs) are a family of animal lectins that show the specific binding activities to l-rhamnose or d-galactose and mainly isolated from eggs and ovary cells of fishes and invertebrates [39, 56, 57, 126]. Sea urchin egg lectin (SUEL) is the first example of isolated and sequenced RBL family [11]. SUEL forms a homodimer composed of two identical subunits, which consist of 105 amino acid residues including single CRD, via intersubunit disulfide bond, resulting in the hemagglutinating activity with bivalent binding properties. To date, the RBL family has been found in over 20 species of fish, which located specifically in oocytes, ovaries, and skin mucus [52, 54, 56–63]. RBLs have been also found in the mantle of penguin wing oyster [39], and ascidians [32, 33]. Except for the reproductive cells including oocyte and egg, RBLs are mainly located in the tissue related to the immune system such as mucous cells of gill, goblet cells of intestine, spleen, thrombocyte, lymphocyte, monocyte, and neutrophil [127, 128]. Moreover, RBLs were isolated from spores of the microsporidian fish parasite, *Loma salmonae*, which was located in gill tissue [129] and *Glugea plecoglossi* from ayu eggs [61], respectively. Thus, it is possible that RBLs participate in the self-defense mechanisms. In fact, the receptor of RBL from amago (*Oncorhynchus rhodurus*) was expressed on the peritoneal macrophage after inflammatory stimulation [130], and RBLs from grass carp (*Ctenopharyngodon idellus*) roe induced a dose-dependent increase in phagocytic activity of seabream macrophage [131].

## 6. Structural Characterization of RBLs: Primary Structures and Classification

Three RBLs, named CSL1, CSL2, and CSL3, have been isolated as rhamnose-binding lectins from chum salmon (*Oncorhynchus keta*) eggs [41]. The amino acid sequences among CSLs show the 42–52% identities, while CSLs show the 94 to 97% sequence identities compared to corresponding three RBLs, STL1, STL2, and STL3, from steelhead trout *(Oncorhynchus mykiss*) eggs, respectively. Moreover, CSL1, CSL2 and CSL3 are composed of 4, 18, and 2 subunits via noncovalent binding, respectively. 

**Figure 3:**
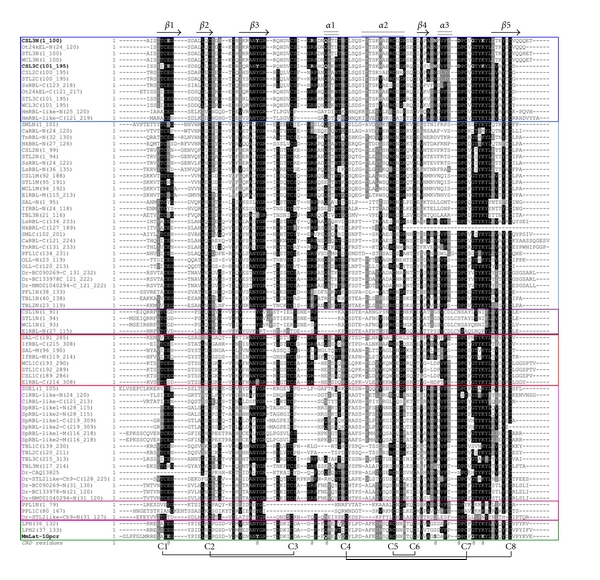
Aligned amino acid sequences of RBL-CRDs. Multiple alignment was achieved using the CLUSTAL X program. Secondary structural elements are shown as cylinders (*α* helix, *α*1–*α*3) and arrows (*β* strand, *β*1–*β*5). Disulfide-bond paring of eight half-cystine residues (C1–C8) is indicated at the bottom. Bold-faced amino acids indicate the residues involved in the carbohydrate binding. The sequence data were obtained from the Entrez protein sequence database including SwissProt, PIR, PRF, and PDB. The CRDs located in the N-terminal, middle, and C-terminal regions are represented by N, M, and C, respectively. CSL: chum salmon (*Oncorhynchus keta*) egg lectin, OLL: shishamo smelt (*Osmerus lanceolatus*) egg lectin, PFL: ponyfish (*Leiognathus nuchalis*) egg lectin, PPL: penguin wing oyster (*Pteria penguin*) lectin, SAL: catfish (*Silurus asotus*) egg lectin, SFL: ayu (*Pleacoglossus altivelis*) egg lectin, SML: Spanish mackerel (*Scomberomorous niphonius*) egg lectin, STL: steelhead trout (*Oncorhynchus mykiss*) egg lectin, SUEL: sea urchin (*Anthocidaris crassispina*) egg lectin, TBL: far east dace (*Tribolodon brandti*) egg lectin, WCL: white spotted charr (*Salvelinus leucomaenis*) egg lectin. Bs: *Botryllus schlosseri*, Ca: snakehead (*Channa argus*), Ci: Ciona intestinalis, Dr: zebrafish (*Danio rerio*), El: northern pike (*Esox lucius*), Hk: spotted seahorse (*Hippocampus kuda*), Hm: *Hydra magnipapillata*, If: blue catfish (*Ictalurus furcatus*), Ls: humphead snapper (*Lutjanus sanguineus*), Mm: house mouse (*Mus musculus*), Ot: small-eared galago (*Otolemur garnettii*), Sp: *Strongylocentrotus purpuratus*, Ss: Atlantic salmon (*Salmo salar*), Tn: green pufferfish (*Tetraodon nigroviridis*).

**Figure 4:**
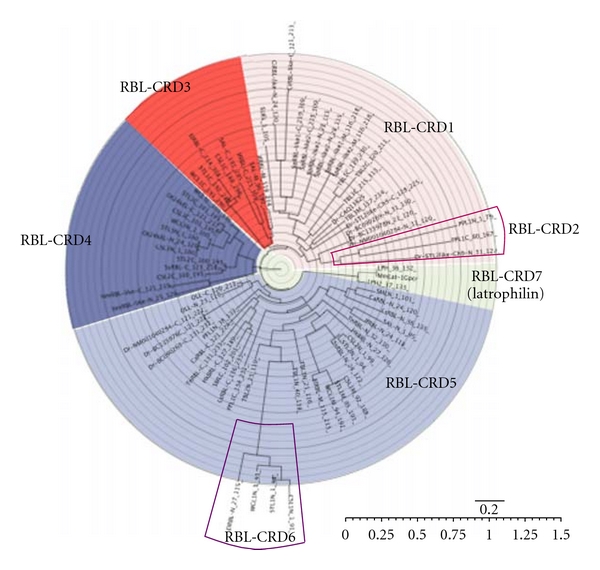
Phylogenetic tree of CRDs of RBL family lectins. A phylogenetic tree was constructed by the neighbor-joining algorithm based on an evolutionary distance matrix constructed by Kimura*'*s method.

Most RBLs are composed of two or three tandem-repeated CRDs, which consist of about 95 amino acid residues, and share the conserved topology of four disulfide bonds.  Figure 3 shows the aligned amino acid sequences of various RBL-CRDs. The disulfide bond pairings of RBLs have been determined for Spanish mackerel lectin (SML) by protein sequencing combined with peptide mapping [59] and for SEL24K from Chinook salmon by matrix-assisted laser desorption/ionization (MALDI) mass-spectrometry [132], respectively. Each RBL-CRD had the same disulfide bonding patterns: Cys(1)–Cys(3), Cys(2)–Cys(8), Cys(4)–Cys(7), and Cys(5)-Cys(6) (Figure 3). Furthermore, two characteristic peptide motifs, -(AN)YGR(TD)-(YGR-motif) and -DPCX(G)T(Y)KY(L)-(DPC-motif), which are located at the N- and C-terminal regions in each domain, respectively, are conserved in almost RBL-CRDs. However, the structural variations for S–S bonds and motifs are observed in PPL and N-terminal CRDs of CSL1, STL1, WCL1, and ElRBL (Figure 3). Previously, RBLs have been classified into five groups (Types I to V) based on their domain structures and the hemagglutination activity against human erythrocytes and sugar specificity against lactose (Table 3) [133]. Type I is composed of three tandemly repeated domains. Type II has two tandem-repeated domains with an extra domain. Types III and IV have two tandem-repeated domains, but they have different hemagglutination activity and sugar specificity. Type V has only one RBL domain and exits in a homodimer with a disulfide linkage between subunits. On the other hand, the phylogenetic tree constructed from the amino acid sequences of CRDs derived from several RBLs revealed that RBL-CRD can be classified into seven groups, RBL-CRD1 to RBL-CRD7, as shown in Figure 4. Based on their structural features of RBL-CRDs compositions, RBLs can be classified into 13 subgroups (Ia to V) (Table 3). The subunit of CSL1 is composed of 286 amino acid residues with three tandemly repeated domains (Type II), while the subunits of CSL2 and CSL3 are composed of 195 amino acid residues with two tandem-repeated domains (Type III). 

**Figure 5:**
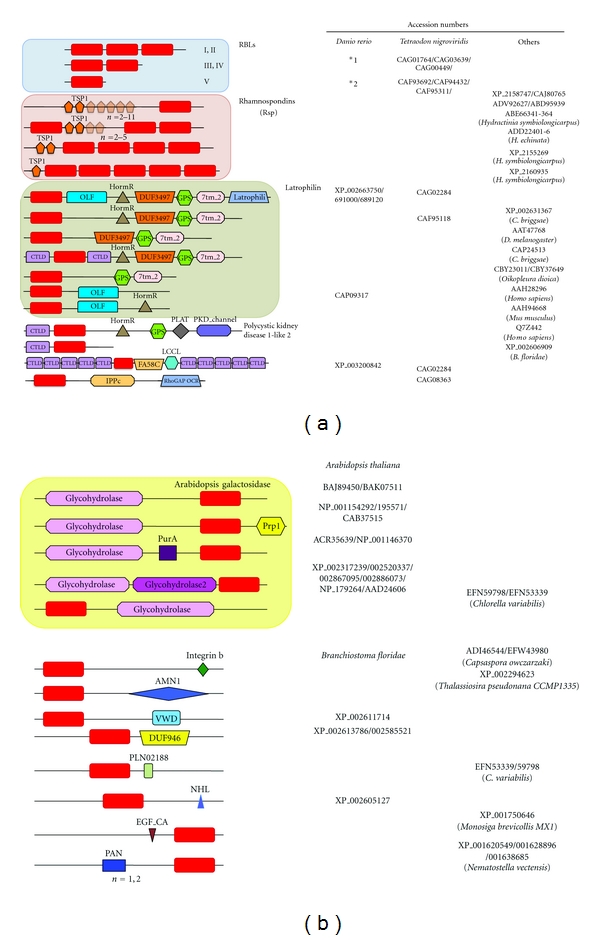
Domain architecture of RBL superfamily. The proteins with similar domain architectures have been identified using the Conserved Domain Architecture Retrieval Tool (CDART) [124].

Furthermore, recent studies including genome-wide screening revealed several variations in the RBL families. It was found that the genes containing the distinctive structural motif of RBL-CRDs broadly distributed in almost all the animals including invertebrate *(Hydra magnipapillata, Hydractinia echinata, Strongylocentrotus purpuratus, Nematostella vectensis, Caenorhabditis remanei, Triatoma dimidiata), Chordates (Ciona intestinalis, Botryllus schlosseri, Branchiostoma floridae),* and vertebrate including bony fish such as *Danio rerio, Oncorhynchus mykiss*,* and so forth,* amphibian* Xenopus tropicalis* and mammalians such as* Mus musculus, Rattus norvegicus, Homo sapiens*,* and so forth,* and also in the bacterium (*Flavobacterium*) and plants (*Arabidopsis thaliana*, *Medicago truncatula*) when the genomic database was retrieved. For example, the RBL homologues have been reported as integrated domains involved in the ligand binding in membrane receptors such as polycystic kidney disdase-1-like (PKD-1) [134], axon guidance receptor EVA-1 [135], HuC21orf63 [136], and the adhesion-class G-protein-coupled receptor latrophilin (LPHN) [137, 138].  Figure 5 summarized the domain architectures of RBL superfamily proteins that contained RBL-CRDs in their sequences as a domain structure. (TSP1: thrombospondin-type 1 repeats, OLF: olfactomedin-like domain, HormR: hormone receptor domain, GPS: G-protein-coupled receptor proteolytic site domain, 7tm_2: 7 transmembrane receptor, latrophili: latrophilin cytoplasmic C-terminal region, CTLTD: C-type lectin domain. PLAT: polycystic kidney disease protein-1-like 2, PKD_channel: polycystin cation channel, FAS8C: coagulation factor 5/8 C-Terminal domain, discoidin domain, LCCL: limulus-clotting factor C, Coch-5b2, and Lgl1-lectin domain, IPPc: inositol polyphosphate phosphatase, catalytic domain homologues, RhoGAP_OCR: GTPase-activator protein for Rho-like Small GTPases in oculocerebrorenal syndrome of Lowe-1-like protein, Prp1: proline-rich protein 1, PurA: adenylosuccinate synthase, AMN1: antagonist of mitotic exit network protein 1, VWD: von Willebrand factor domain, NHL: Ncl-1, HT2A, and Lin-41 proteins, PAN module: plasminogen/hepatocyte growth factor-Apple domains of the plasma prekallikrein/coagulation factor XI-Nematode proteins module, *1: AAH90269/AAI22308/50383/CAM56745/56747/CAX13501/NP_001035384/001038891/XP_692814/001922851/003199967/003200629/706941/003200654/003200655, *2: AAI22302/50374/51864/51941/54461/54558/55629/62646/62650/CAK11496-11501/11504/11506/11509/11515/CAM56419-56422/56424/56426/56637/56466/56467/56470-56473CAP09587/09516/CAQ13825/14198-14199/NP_001038882/001082844/001082910/001082869/001093874/001093910/001096104/001098618/001103190/001103311/001103315/001103334/001103858/001104195/001108359/001103581/001103589/001095862/001103856/001138280/001128340/001153845/002663370/002663371/002666350/XP_003199229/003199230/002663369/692138/003200629/706941/003200654-655/003200654-655/003201119/003201121/003201123/003201136/002666347/003201138-39/002667144/002666349/001333550. Beside the RBLs (Types I–V), several groups are classifiable as an RBL superfamily: the immune recognition molecules, rhamnospondins (Rsps), LPHNs, *Arabidopsis *galactosidases, and others as shown in Figure 5. Rsp gene has been identified in colonial hydroid *Hydractinia symbiolongicarpus* and was found to encode a secreted modular protein of 726 amino acids composed of N-terminal serine-rich domain, eight tandem-repeated thrombospondin type 1 repeats (TSRs), and C-terminal RBL-CRD [139]. Rsps have diversified by gene duplication and predicted to act as immune recognition molecules from the evidence of gene structure and their expression profiles in the polyp's hypostome, which face to the external environment and pathogen. On the other hand, *Caenorhabditis elegans* EVA-1 forms a complex with SAX-3/roundabout (Robo) receptor and functions as a coreceptor for shiga-like toxin 1 (SLT-1)/slit proteins in guiding cell and axon migrations [135]. Furthermore, human C21orf63, an EVA-1 ortholog, has been identified from the Down's syndrome project [136] and reported to have specific affinity to heparin. 

LPHNs are synaptic Ca^2+^-independent *α*-latrotoxin (LTX) receptor, a novel member of the secretin family of G-protein-coupled receptors containing seven transmembrane regions as well as long N-terminal extracellular sequences containing a 19-amino acid signal peptide, and a serine/threonine-rich glycosylation region (Figure 5) [137]. LTX is a component of the venom of the black widow spider (*latrodectus mactans*) and stimulates exocytosis of *γ*-aminobutyric acid- (GABA-) containing presynaptic vesicles via interaction with LPHN. Recently, LPHN3, which is the most brain-specific LPHN, has been reported to be involved in the pathogenesis of attention-deficit/hyperactivity disorder [138]. Thus, the RBL-CRDs are diversified and widely distributed in the functional proteins as unique structural motifs. Similar examples for the domain architecture of proteins including membrane receptors have been reported in other lectin families such as CTLD [74–79] and F-type lectin superfamilies [122, 123]. 

## 7. Gene Structure of RBL Family

More recently, cloning and characterization of a gene for snakehead lectin (SHL) from *Channa argus* and its promoter region have been reported (Genebank accession nos.: EU693900) [140]. SHL gene, which consists of 2,382 bp from the transcription initiation site to the end of 3′ untranslated region (UTR) and includes two tandem RBL-CRDs with 35% identity, contains nine exons and eight introns. The first 40 bp of exon 1 is 5′UTR, and the signal peptide is encoded by exons 1 and 2. The N-terminal CRD is encoded by exons 3, 4, and 5, and C-terminal CRD is encoded by exons 6, 7, and 8. Exon 9 includes the C-terminal region of SHL and 3′UTR. These suggest that RBL-CRDs are located in three exons; respectively, and RBL may be diverged and evolved by gene duplication and/or exon shuffling. The 5′ flanking regions contained some unique consensus sequence for the nuclear factor of interleukin 6 (NF-IL6) and IFN-*γ* activation sites. 

On the other hand, Rsp gene was predicted to encode a secreted protein of 726 amino acids composed of a signal peptide, an N-terminal serine-rich domain (SRD), eight TSRs, and a RBL-CRD at C-terminal region, consisting of 13 exons and 12 introns [139, 141]. However, RBL-CRD of *Rsp* was located in only single exon (Exon 12), suggesting that the gene structure of Rsp RBL-CRD is different from that of SHL RBL-CRD. Whole genome sequences have been determined for several living organisms including marine organisms such as zebrafish, *Danio rerio*, of which data can be available and allow us to establish the full inventory of any particular gene family in the genome. Thus, searching the zebrafish database with RBL-CRD sequences revealed that the genes encoding RBLs with tandem-repeated CRDs (Types I to IV) were located in chromosomes 9, 19, and 21 (Table 4). Furthermore, it was found that several genes encoding single RBL-CRD proteins, almost all of which physiological functions are largely unknown, are located in chromosomes 2, 9, 23, and especially in chromosome 22 (Table 4), although the genes for latrophilins and C21orf63 homolog were located in chromosomes 22 and 14, respectively. 

## 8. Sugar-Binding Specificities and Physiological Functions of RBLs

Sugar-binding specificities of CSLs were investigated thoroughly by frontal affinity chromatography (FAC) using 100 kinds of sugar chains including *N*-linked and glycolipid-type glycans [142]. Interestingly, all of CSL1, CSL2, and CSL3 showed the high specific binding activity against globotriaosyl ceramide (Gb3; Gal*α*1-4Gal*β*1-4Gal*β*1-Cer also known as CD77), which is located in lipid raft and upregulated through immune responses [143] and is also known as the functional receptor for various toxins such as Shiga toxin (Stx) [144], regardless of their low sequence homologies (42–52%) and different oligomeric structures. 

CSLs induced proinflammatory cytokines, including IL-1*β*1, IL-1 *β*2, TNF-*α*1, TNF-*α*2, and IL-8, by recognizing Gb3 on the surface of the peritoneal macrophage cell line (RTM5) from rainbow trout and an established fibroblastic-like cell line (RTG-2) from gonadal tissue of the fish [142]. RBL from catfish, SAL, has also induced the alterations of gene expression in Burkitt's lymphoma cells [145]. Furthermore, CSLs showed the cytotoxicity against Gb_3_-displaying Caco-2 and Lovo cells via an apoptotic pathway through the recognizing of Gb_3_ on the cell surfaces in a dose-dependent manner, while it was not observed with DLD-1 and HCT-15 human colonic tumor cell lines lacking Gb_3_ [125]. 

RBLs from fish eggs such as STLs and CSLs also interacted and agglutinate Gram-negative and Gram-positive bacteria by recognizing the cell-surface lipopolysaccharides and lipoteichoic acid, respectively, [17, 146]. On the other hand, PPL, an RBL from *Pteria penguin* pearl shell, also showed the strong agglutinating activity against some Gram-negative bacteria such as *Escherichia coli* by recognizing lipopolysaccharides in the presence of the high concentration (500 mM) of NaCl, in which condition the oligomerization of PPL was induced, although its carbohydrate-binding specificity was quite different from that of CSLs; PPL binds to d-galactose but not l-rhamnose [39]. RBLs can recognize the O-antigen, which is the immunodominant structure exposed to the environment and is highly variable among bacterial strains, via diverse carbohydrate recognition ability. RBLs bind to glycolipids and glycoproteins of the microsporidian fish pathogens [61, 129], and the RBL receptor was expressed on peritoneal macrophages of fishes after an inflammatory stimulation [130, 147]. More recently, it was found that CSLs induced the production of radical oxygen species (ROS) in RTM5 cells in a dose-dependent manner. This effect was not inhibited by l-rhamnose or dl-threo-1-phenyl-2-palmitoylamino-3-morpholino-1-propanol (PPMP), an inhibitor of glucosyl ceramide synthesis, suggesting that carbohydrate recognition domains of CSLs were not involved in the respiratory burst of RTM5 cells. 

Thus, CSLs are multifunctional lectins through binding to the carbohydrate such as Gb_3_ on cells and l-rhamnose/d-galactose residue of O-antigen of lipopolysaccharides. However, this carbohydrate-binding specificity of RBL leads to the interesting questions of how CSL can strongly recognize the different sugars such as Gb3, l-rhamnose, d-galactose, all of which common structural features are the orientation of anomeric hydroxy groups at C2 and C4.

## 9. Structural Characterization of RBL-CRDs

**Figure 6:**
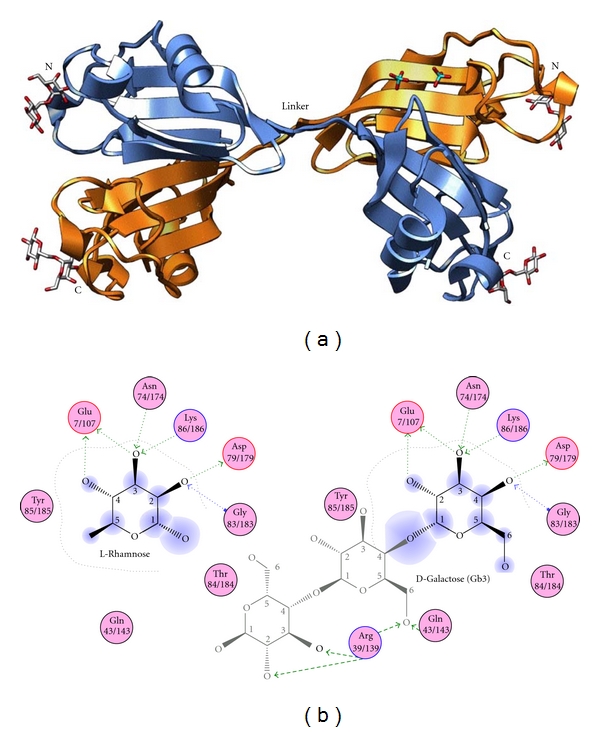
Crystal structure of CSL3. (a) Pseudotetrameric structure of CSL3 dimer (PDB code: 2z×3) [125]. Each carbohydrate-binding domain is differently colored. The bound melibioses and phosphates are shown in stick models. (b) Comparison of the carbohydrate-binding manners of CSL3 complex with l-rhamnose (left) and Gb3 (right).

More recently, the highly ordered structure of CSL3 composed of two subunits of 20 kDa has been determined at 1.8 Å resolution [146]. The homodimer of CSL3 revealed a kinked dumbbell shape, in which two lobes are connected through linkers composed of two 5-residue peptides (-QQQET-) (Figure 6(a)). Each lobe seems to be a single globular protein with a pseudo-twofold axis and includes two antiparallel *β* sheets with two (*β*2 and *β*4) and three (*β*1, *β*3, and *β*5) strands and three helices (*α*1-3) (Figure 6(a)). The N- and C-terminal domains, both of which share 35% sequence identity with the RBL-CRD for mouse latrophilin-1 (LPHN-1), their folds were similar each other and superimposed on the RBL domain of LPHN-1, which has been recently reported [148], with rmsds of 1.4 and 1.5 Å for 94 Ca atoms, respectively. These RBL domains adopt a unique *α*/*β* fold with long structured loops involved in monosaccharide recognition.

It was found that the monosaccharide (rhamnose) or nonreducing end residues (Gal1 of melibiose and Gb_3_) share the same conserved primary binding sites in CSL3 (Glu7/107, Tyr27/127, Lys86/186, and Gly83/183) and LPHN-1 (Glu42, Tyr63, Lys120, and Gly117), respectively, (Figure 6(b)). Asn74/174 and Asp79/179 are additionally used in the primary site in CSL3. The melibiose and Gb_3_ complex structures revealed the oligosaccharide recognition mechanism of RBL. The Arg39/139 and Gln43/143 residues of CSL3, which bind to the carbohydrate in the 2nd and 3rd sites, are the key residues in determining specificity for oligosaccharides, Gb3. The total numbers of hydrogen bonds between CSL3 and rhamnose, melibiose, and Gb_3_ are 7, 8, and 10, respectively, which are consistent with the observed high affinity (*K*_*d*_ = 2.6 × 10^−5^ M) of CSL3 to Gb_3_.

Interestingly, RBLs can bind to l-rhamnose and nonreducing d-galactose moiety of melibiose and Gb_3_ at the same binding site. These specific and characteristic binding abilities can be explained by the recognizing mechanisms involved in the hydrogen bonds between O_2_, O_3_, and O_4_ atoms of monosaccharide and the side chains of Glu7/107, Asn74/174, Asp79/179, Lys86/186, and the main chain of Gly83/183 of CSL3; that is, Glu7/107 forms hydrogen bond with O_4_ atom of l-rhamnose, which correspond to O_2_ of inverted d-galactose, while Gly83/183 forms hydrogen bond with O_2_ atom of l-rhamnose and O_4_ of inverted d-galactose form, respectively, (Figure 6(b)). These carbohydrate recognition mechanisms of lectins for the inverted carbohydrates were also found in the case for F-type lectins, which bind to both *α*-l-fructose and 3-O-methyl-d-galactose [122].

## 10. Perspectives

Since lectins isolated from marine resources are highly diversified in terms of not only structure but also functional aspects including specific and unique carbohydrate specificities as reviewed in this paper, they can be used for biomedical application as drug delivery system or diagnostic markers. For example, RBL family lectins are useful for diagnosis of pathological condition involved in Gb3 ceramide such as Burkitt's lymphoma having high malignancy. Furthermore, RBLs showed the physiological functions independent of carbohydrate-recognition ability such as ROS-inducing activities although its molecular mechanism has not yet been clarified. More recently, novel calcium-dependent mannose-binding lectin, intelectin, which is structurally identical to the intestinal receptor for lactoferrin and contained fibrinogen-related domain, has been identified from the skin mucus of catfish *Silurus asotus* [55]. Thus, there are a large and growing number of diversified lectins in marine resources. Further study will be necessary to elucidate the detailed structure-activity relationships of diversified marine lectins and to develop the potent therapeutic drugs.

## Figures and Tables

**Table 1:** Lectins from marine organisms.

Organism	Lectins	Binding specificity	Lectin family	References
[Protozoan]				
Sponge				
(*Aphrocallistes vastus*)	LECCI	d-Galactose	C-type	[93]
(*Axinella polypoides*)	AP-I/II/III/V	d-Galactose	Ricin type	[94]
	AP-IV	hexuronic acid		
(*Geodia cydonium*)	GCLT1	LacNAc	Galectin	[95]
(*Ptilota filicina*)	PFL	d-Galactose		[96]

[Mollusk]				
*Tridacna*				
(*Tridacna maxima*)	tridacin	N-acetyl galactosamine	C-type	[37]
Abalone				
(*Haliotis laevigata*)	PLC	d-Galactose/d-Mannose	C-type	[38]
Penguin wing oyster				
(*Pteria penguin*)	PPL	d-Galactose	**RBL**	[39]

[Arthropod]				
Barnacle				
(*Megabalanus rosa*)	BRA-1~ 3	d-Galactose	C-type	[8, 9]
(*Balanus rostratus*)	BRL	d-Galactose	C-type	[10]
Horseshoe crab				
(*Tachypleus tridentatus*)	TL-1	LPS (KDO), LTA	6x *β*-propeller/Tectonin	[19]
	TL-2	GlcNAc/GalNAc, LTA	5x *β* propeller	[20–22]
	TL-3	LPS (*O*-antigen)	F-type	[23]
	TL-4	LPS(*O*-antigen), LTA,		[24]
	TLs-5	Fucose	Ficolin	[25, 26]
	TL-P	*N*-acetyl group	6x *β*-propeller/Tectonin	[27]
	GBP/PAP/LBP	GlcNAc		[28]

[Echinoderm]				
Sea cucumber				
(*Cucumaria echinata*)	CEL-I/CEL-II	GlcNAc	C-type	[13, 14]
	CEL-IV	GlcNAc-Galactose	C-type	[13, 15]
	CEL-III	d-Galactose and so forth	Ricin type	[13, 16]
(*Stichopus japonicus*)	SPL-1	uronic acid	C-type	[17]
	SPL-2	LacNAc and so forth	C-type	[17]
	SJL-1	d-Galactose	C-type	[18]
Urchin				
(*Anthocidaris crassispina*)	SUEL	d-Galactose	**RBL**	[11]
	Echinoidin (ECH)	d-Galactose	C-type	[12]
Starfish				
(*Asterina pectinifera*)		LacNAc	C-type	[97]

[Protochordate]				
Ascidian				
(*Polyandrocarpa misakiensis*)	TC14	d-Galactose	C-type	[31]
(*Botryllus schlosseri*)	Bs RBL-1~5	l-Rhamnose	**RBL**	[32, 33]
(*Halocynthia roretzi*)	P36	d-Galucose	C-type	[34]
	P40-P50		ficolin	[35]
	41KD	d-Glactose	C-type (ficolin)	[36]

[Fishes]				
Japanese eel				
(*Anguilla japonica*)	AJL-1	*β*-Galactoside	Galectin	[40]
	AJL-2	Lactose	C-type	[41]
	eCL-1/eCL-2	Lactose	C-type	[42]
European eel				
(*Anguilla anguilla*)	AAA	Fucose	F-type	[43]
Electric eel				
(*Electrophorus electricus*)	Electrolectin	Lactose and so forth	Galectin	[44]
Conger eel				
(*Conger myriaster*)	Congerin I	Lactose and so forth	Galectin	[45, 46]
	Congerin II	Lactose and so forth	Galectin	[45, 47]
	Congerin P	Lactose/mannose	Galectin	unpublished
	conCL-s	mannose	C-type	[48]
Windowpane flounder				
(*Lophopsetta maculate*)		d-Galactose	Galectin	[49]
Zebrafish				
(*Danio rerio*)	Drgal1-L1–L3	LacNAc	Galectin	[50]
Shishamo smelt				
(*Osmerus lanceolatus*)	OLABL	d-Galactose	C-type	[51]
	OLL	l-Rhamnose	**RBL**	[52]
Catfish				
(*Arius thalassinus*)		d-Galactose	Galectin	[53]
(*Silurus asotus*)	SAL	l-Rhamnose	**RBL **	[54]
	saIntL	d-Mannose	intelectin	[55]
Steelhead trout				
(*Oncorhynchus mykiss*)	STL-1–3	l-Rhamnose	**RBL**	[56, 57]
Chum salmon				
(*Oncorhynchus keta*)	CSL-1–3	l-Rhamnose	**RBL**	[58]
White-spotted charr				
(*Salvelinus leucomaenis*)	WCL-1, 3	l-Rhamnose	**RBL**	[59]
Spanish mackerel				
(*Scomberomorus niphonius*)	SML	l-Rhamnose	**RBL**	[60]
Sweet fish (ayu)				
(*Plecoglossus altivelis*)	SFL	l-Rhamnose	**RBL**	[61]
Ponyfish				
(*Leiognathus nuchalis*)	PFL-1,2	l-Rhamnose	**RBL**	[62]
Far-East dace				
(*Tribolodon brandti*)	TBL-1–3	l-Rhamnose	**RBL**	[63]
Striped bass				
(*Morone saxatilis*)		Fucose	F-type	[64]
Sea bass				
(*Dicentrarchus labrax*)	DlFBL	Fucose	F-type	[65]
Japanese sea perch				
(*Lateolabrax japonicus*)	JspFL	Fucose	F-type	[66]
Pufferfish				
(*Fugu rubripes*)	Pufflectin	d-Mannose	Lily-type	[67]
Scorpionfish				
(*Scorpaena plumieri*)	plumieribetin	d-Mannose and so forth	Lily-type	[68]
Carp				
(*Cyprinus carpio*)	carpFEL	GlcNAc	6x *β*-propeller/Tectonin	[69]

AAA: *Anguilla anguilla* agglutinin; AJL: *Anguilla japonica* lectin; BRA: *Megabalanus rosa* lectin; BRL: *Balanus rostratus* lectin; CEL: *Cucumaria echinata* lectin; CSL: chum salmon lectin; GCLT1: *Geodia cydonium* lectin; OLABL: *Osmerus lanceolatus* asialofetuin-binding lectin; OLL: *Osmerus lanceolatus* lectin; PFL: ponyfish lectin; PLC: perlucin; PPL: *Pteria penguin* lectin; SAL: *Silurus asotus* lectin; SFL: sweet fish (ayu) lectin; SJL: *Stichopus japonicus* lectin; SML: Spanish mackerel lectin; SPL: sea cucumber plasma lectin; STL: Steelhead trout lectin; SUEL: sea urchin egg lectin; TBL: *Tribolodon brandti* lectin; WCL: white spotted charr lectin; KDO: 2-keto-3-deoxyoctonate; LPS: lipopolysaccharide; LTA: lipoteichoic acid; GalNAc: N-acetyl galactosamine; GlcNAc: N-acetyl glucosamine; LacNAc: N-acetyl lactosamine.

**Table 2:** Zebrafish galectin-related genes.

	Length		Chromosome	Exons	Gene ID*
	Transcripts (bp)	Protein (AA)			
Drgal1 (proto)					
lgals1 (Gal1-L1)	883	134	3	4	326706
lgals2a (Gal1-L2)	850	134	3	4	405830
lgals2b (Gal1-L3)	545	118	6	4	393486
si:ch211-10a23.2	1951	149	13	4	567812

Drgal3 (chimera)					
zgc112492 (Gal3)	1993	561	3	5	550351
lgals3l (Gal3-like)	958	228	17	6	325599
lgals3bpa (Gal3-like)	2005	567	3	5	677742
lgals3bpb (Gal3-like)	1983	572	3	5	405809
si:ch211-67f24.5	1925	369	3	5	125833577

Drgal4 (tandem)					
LOC567193	3981	—	18	25	567193

Drgal8 (tandem)					
LOC100334749	864	287	17	7	100334749
si:ch211-199I3.2	537/402	153/133	20	9	368889

Drgal9 (tandem)					
zgc92326 (Gal9-L1)	2591	321	15	11	327284
lgals9l1	1075	310	15	10	337597
LOC100148547	1017	282	15	7	100148547
zgc171951	1453	280	15	7	100124603

Others					
Grifin (zgc92897)	684	139	3	4	445036
GRP (zgc136758)	3518	164	1	5	723995
GRP (si:ch211-101n13.9)	438	145	13	2	563573
LOC100535066	3984	1327	18	29	100535066
c7orf23 (zgc:112101)	1011	154	18	3	550443

*Gene ID: ID numbers in NCBI's database for gene-specific information [98].

**Table 3:** Classification of RBL family lectins.

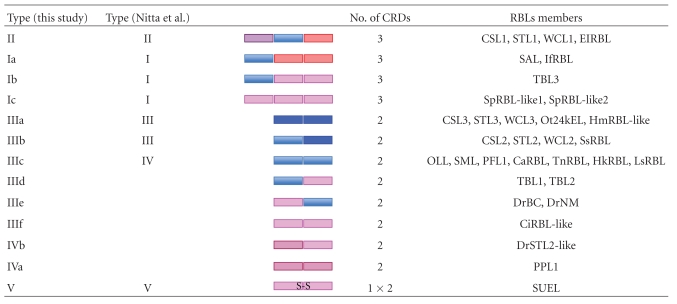

**Table 4:** Zebrafish RBL-related genes.

	Length		Chromosome	Exons	Gene ID*
	Transcripts (bp)	Protein (AA)			
Type I/II (3CRDs)					
LOC100536087	1063	274	19	10	100536087
LOC555549	1144	297	19	10	555549

Type III/IV (2CRDs)					
Zgc136410	878	222	21	9	449685
LOC100536640	978	233	19	9	100536640
LOC100535118	715/868	167/209	9	8/9	100535118
LOC100536038	1050	272	19	9	100536038
LOC100536135	— (pseudo)	—	19	9	798039

1CRD					
LOC100333730	654/618	119/137	9	6	100333730
LOC100301575	665	128	22	6	100301575
LOC798111	668	128	22	6	798111
LOC100535660	469	129	22	7	100535660
MGC171851	677	128	22	6	798039
LOC100331690	815	186	22	7	100331690
LOC100537146	837	188	22	7	100537146
LOC100537102	804	179	22	7	100537102
LOC100536958	821	188	22	7	100536958
LOC794480	750	137	23	9	794480

Others					
Latrophilin-3-like	609	131	22	6	445036
Latrophilin 2	669	128	22	7	94733430
DKEYP-98A7.10	620	168	22	7	100126138
LOC792919	652	190	2	6	792919
C21orf63 homolog	1189	380	14	7	100537109

*Gene ID: ID numbers in NCBI's database for gene-specific information [98].

## Abbreviations

AAA: *Anguilla japonica* agglutinin
AFPs: Antifreeze proteins
ASGR: Asialoglycoprotein receptor
CN-V: Cyanovirin
Con I: Congerin I
Con II: Congerin II
CRD: Carbohydrate-recognition domain
CSL: Chum salmon lectin
CTLs: C-type lectins
CTLD: C-type lectin domain
DC-SIGN: Dendritic cell-specific intercellular adhesion molecule-3-grabbing nonintegrin
EC50: Half maximal effective concentration
ES: Embryonic stem
FACT: Frontal affinity chromatography technique
GABA: *γ*-Aminobutyric acid
GRFT: Griffithsin
GRPs: Galectin-related proteins
HIV: Human immunodeficiency virus
iPS: Induced pluripotent stem
LPHN: Latrophilin
LTX: *α*-Latrotoxin
MALDI: Matrix-assisted laser desorption/ionization
MBP: Mannose-binding protein
MES: 2-(*N*-Morpholino) ethane sulfonic acid
PKD-1: Polycystic kidney disdase-1 like
PPL: *Pteria penguin* lectin
PSP: Pancreatic stone protein
RBL: Rhamnose-binding lectin
Rsp: Rhamnospondins
SAL: *Silurus asotus* lectin
SHL: Snakehead lectin
SLT-1: Shiga-like toxin 1
SML: Spanish mackerel lectin
STL: Steelhead trout lectin
SUEL: Sea urchin egg lectin
TSRs: Thrombospondin-type-1 repeats.
